# Altered features and increased chemosensitivity of human breast cancer cells mediated by adipose tissue-derived mesenchymal stromal cells

**DOI:** 10.1186/1471-2407-13-535

**Published:** 2013-11-09

**Authors:** Lucia Kucerova, Svetlana Skolekova, Miroslava Matuskova, Martin Bohac, Zuzana Kozovska

**Affiliations:** 1 Laboratory of Molecular Oncology, Cancer Research Institute, Slovak Academy of Sciences, Vlarska 7, 833 91, Bratislava, Slovakia; 2 Department of Plastic, Aesthetic and Reconstructive Surgery, University Hospital, Bratislava, Slovakia; 3 Institute of Histology and Embryology, Faculty of Medicine, Comenius University, Bratislava, Slovakia

**Keywords:** Adipose tissue-derived mesenchymal stromal cells, Human breast cancer, Chemoresistance, Proliferation, Epithelial-to-mesenchymal transition, Cytokine profile

## Abstract

**Background:**

Mesenchymal stromal cells (MSCs) represent heterogeneous cell population suitable for cell therapies in regenerative medicine. MSCs can also substantially affect tumor biology due to their ability to be recruited to the tumor stroma and interact with malignant cells via direct contacts and paracrine signaling. The aim of our study was to characterize molecular changes dictated by adipose tissue-derived mesenchymal stromal cells (AT-MSCs) and the effects on drug responses in human breast cancer cells SKBR3.

**Methods:**

The tumor cells were either directly cocultured with AT-MSCs or exposed to MSCs-conditioned medium (MSC-CM). Changes in cell biology were evaluated by kinetic live cell imaging, fluorescent microscopy, scratch wound assay, expression analysis, cytokine secretion profiling, ATP-based viability and apoptosis assays. The efficiency of cytotoxic treatment in the presence of AT-MSCs or MSCs-CM was analyzed.

**Results:**

The AT-MSCs altered tumor cell morphology, induced epithelial-to-mesenchymal transition, increased mammosphere formation, cell confluence and migration of SKBR3. These features were attributed to molecular changes induced by MSCs-secreted cytokines and chemokines in breast cancer cells. AT-MSCs significantly inhibited the proliferation of SKBR3 cells in direct cocultures which was shown to be dependent on the SDF-1α/CXCR4 signaling axis. MSC-CM-exposed SKBR3 or SKBR3 in direct coculture with AT-MSCs exhibited increased chemosensitivity and induction of apoptosis in response to doxorubicin and 5-fluorouracil.

**Conclusions:**

Our work further highlights the multi-level nature of tumor-stromal cell interplay and demonstrates the capability of AT-MSCs and MSC-secreted factors to alter the anti-tumor drug responses.

## Background

Breast cancer still remains one of the most common malignancies in women with multiple risk factors [1]. Any solid tumor derived from breast epithelial tissue is supported by tumor stroma – a non-malignant tumor compartment composed from multiple cell types and non-cellular components. The tumor microenvironment creates a complex signaling network which substantially affects tumor biology and therapeutic responsiveness [2,3]. Adipose tissue is the most abundant stromal constituent in the breast and also a rich source of mesenchymal stromal cells (MSCs) which contribute to mammary carcinogenesis [4]. As a fat grafting procedure is frequently used in breast reconstruction, breast contour deformity correction or even in breast augmentation, it also carries potential oncological risk of de novo breast cancer and/or its recurrence [5,6].

The MSCs derived from the adipose tissue (AT-MSCs) share a number of key characteristics with the bone marrow-derived MSCs (BM-MSCs) [7-9]. MSCs from both sources were demonstrated to integrate into tumor-associated stroma and exhibit multiple regulatory functions in the tumor microenvironment [10-12]. Experimental data revealed the capability of BM-MSCs to differentiate into tumor-associated fibroblasts [13-15] and even create a cancer stem cell niche [16] when exposed to tumor-conditioned medium. The interaction of BM-MSCs and breast cancer cells was also shown to promote metastatic spread as a result of bidirectional paracrine signaling [17]. Although the effect on proliferation of the tumor cells was not stimulatory in general, MSCs were shown to promote tumor cell migration, an epithelial-to-mesenchymal transition (EMT), mediate release from the hormone-dependence, and increase chemoresistance in breast cancer cells [18-23]. MSCs-secreted factors increased mammosphere formation and the exosomes from MSCs were sufficient to support the growth of tumor xenografts [24-26]. Taken together these data suggest that BM-MSCs promote breast cancer growth and/or metastatic spread. However, a suppression of the tumor growth by MSCs was reported for the tumor types other then breast; and the role of MSCs in tumor growth remains a matter of further investigations [12,27-29]. Better understanding of the underlying mechanisms might lead to the therapeutic intervention with the aim to increase an antitumor response [30,31]. MSCs themselves can be specifically engineered for the increased tumor-targeting and efficiency of the anti-tumor treatment [32]. The introduction of specific transgene(s) into the AT-MSCs sensitized the breast cancer cells MDA-MB-231 to the chemotherapeutic drug 5FU for *in vitro*[33].

We have previously characterized the effect of AT-MSCs on the proliferation of breast cancer cells; and linked it to the cytokine secretion profile of AT-MSCs [23]. In this study we have focused on the multiple alterations induced in human Her2-positive breast cancer cell line SKBR3 by the AT-MSCs. We have extended our investigation also on the effect of stromal cells on drug responses in the tumor cells. We have observed that the AT-MSCs induced an EMT, decreased proliferation, increased migration and other molecular changes in the SKBR3 cells. We have shown that the AT-MSCs could alter chemosensitivity of the tumor cells.

## Methods

### Cells

Human tumor cell line SKBR3 (ATCC® Number HTB-30™) was used for the study. Tumor cells were maintained in high-glucose (4.5 g/l) DMEM (PAA Laboratories GmbH) containing 10% FBS (Biochrom AG), 10.000 IU/ml penicillin (Biotica, Part. Lupca, Slovakia), 5 μg/ml streptomycin, 2 mM glutamine and 2.5 μg/ml amphotericin (PAA Laboratories GmbH).

For mammosphere cultures, 4×10^4^ EGFP-SKBR3 cells per well were plated in non-adherent 6-well plates (Ultra-low attachments plates, Corning, Amsterdam, NL) in serum free DMEM/F12 medium (GIBCO-Invitrogen BRL) supplemented with 10.000 IU/ml penicillin (Biotica, Part. Lupca, Slovakia), 5 μg/ml streptomycin, 2 mM glutamine, and 2.5 μg/ml amphotericin (Sigma, St. Louis, MO), 10 ng/ml bFGF (Miltenyi Biotec), 10 ng/ml EGF (Miltenyi Biotec), 4 μg/ml heparin (Sigma, St. Louis, MO), 2 μg/ml insulin (Sigma, St. Louis, MO) and B27 supplement (diluted 1:100, Gibco-Invitrogen BRL) and cultivated at 37°C in humidified atmosphere and 5% CO_2_ for 5 days. Specific inhibitors 1.63 μM LY294002 (Sigma, St. Louis, MO) or 0.5 μM SB203580 (Sigma, St. Louis, MO) were added to the MSCs-CM mammosphere medium as indicated.

AT-MSCs were isolated and characterized by immunophenotype and differentiation potential as previously described in [34] (Additional file 1). The AT-MSCs were expanded in low glucose (1.0 g/l) DMEM supplemented with 10% HyClone® AdvanceSTEM™ supplement (Thermo Scientific) and antibiotic/antimycotic mix (10.000 IU/ml penicillin, 5 μg/ml streptomycin, 2 mM glutamine, and 2.5 μg/ml amphotericin). Different isolates were used for the experiments (n = 4), each experiment was run at least twice with each isolate to draw the conclusions. Cells were maintained at 37°C in humidified atmosphere and 5% CO_2_.

Cell-free AT-MSCs conditioned medium (MSCs-CM) was collected from 80–90% confluent cultures after 24 hours of cultivation with fresh tumor cell culture medium or mammosphere culture medium, respectively, and filtered through 0.45 μm filters. Fresh MSCs-CM was always used for the experiments.

### EGFP expression

Stable transduction of SKBR3 to express enhanced green fluorescent protein (EGFP) was done by retrovirus gene transfer as described elsewhere [33]. Transgene incorporation and EGFP expression was confirmed by PCR, reverse transcription coupled PCR and flow cytometric analysis performed on BD Canto II cytometer (Becton Dickinson, USA) equipped with FACS Diva program. FCS Express software was used for evaluation. The identity of SKBR3 and EGFP-SKBR3 cells was further confirmed by sustained expression of epithelial cell adhesion molecule (CD326, ≥98% positivity) verified by flow cytometry with specific antibody anti-EpCAM-PE (Miltenyi Biotec GmbH, Germany). Mouse IgG1-PE (Miltenyi Biotec GmbH, Germany) was used as negative isotype control.

### Analysis of morphological changes in EGFP-SKBR3

Three ×10^5^ EGFP-SKBR3 cells were mixed with 1.5×10^5^ DiI-stained AT-MSCs and cocultured for 5–9 days. For a comparison, EGFP-SKBR3 cells alone were seeded and cell morphology was analyzed by fluorescent microscopy (Axiovert 200, Zeiss, Germany). Alternatively, quadruplicates of 4×10^4^ tumor cells were seeded in MSC-CM or culture medium in 96-well plates. Phase-contrast images were taken in the IncuCyte ZOOM™ Kinetic Imaging System (Essen BioScience, UK). Cell confluence was evaluated by IncuCyte ZOOM™ 2013A software (Essen BioScience, UK) based on the confluence masks as recommended by manufacturer.

### Migration assay

Fifty thousand EGFP-SKBR3 per well were plated in triplicates in ImageLock 96-well plates (Essen BioScience, UK) and let to adhere for 16 hrs. Confluent monolayers were wounded with wound making tool (Essen BioScience, UK), washed twice and supplemented with MSC-CM or culture medium. As indicated, medium was supplemented with receptor-tyrosine kinase inhibitors 150 nM Pazopanib, 250 nM Sorafenib or 200 nM Sunitinib (inhibitors kindly provided by National Cancer Institute, Bratislava). Images were taken every two hours for next 72 hrs in the IncuCyte ZOOM™ Kinetic Imaging System (Essen BioScience, UK). Cell migration was evaluated by IncuCyte ZOOM™ 2013A software (Essen BioScience, UK) based on the relative wound density measurements and expressed as means of three independent experiments run in triplicates ± SD.

### Gene expression analysis

EGFP-SKBR3 tumor cells were cultured with or without MSC-CM for 6 days with everyday medium replenishment. Total RNA was isolated from 5×10^6^ EGFP-SKBR3 cultured with or without MSC-CM. Cultured cells were collected by trypsinization, RNA isolated by NucleoSpin® RNA II (Macherey-Nagel) and treated with RNase-free DNase (Qiagen, Hilden, Germany). Total RNA was subjected to control PCR to confirm the absence of genomic DNA contamination. RNA was reverse transcribed with RevertAid™ H minus First Strand cDNA Synthesis Kit (Fermentas, Hanover, MD). 200 ng of cDNA was amplified in standard PCR performed in 20 μl 1x PCR master mix (Fermentas, Canada) with 0.5 μl respective specific primers (20 pmol/μl) and DNase free water (Fermentas, Canada) in DNA Engine Dyad™ Peltier Thermal Cycler (MJ Research, UK) with pre-set amplification profile and horizontal electrophoresis was used for detection of amplicons. Each reaction was run with appropriate no template controls and negative control (RNA template without reverse transriptase). Primer sequences were listed in Additional file 2.

Quantitative PCR was performed in 1 × ABsolute™ QPCR SYBR® Green Mix (ABgene, Surrey, UK), 0.16 μM primers and 200 ng of template cDNA on Bio-Rad CFX96™ and analyzed by Bio-Rad CFX Manager software version 1.6. Relative gene expression change was calculated according to ΔΔCt method. GAPDH and HPRT1 gene expression was taken as endogenous reference. Analysis was performed twice in triplicates and data expressed as means ± SD.

### Multiplex and SDF-1α secretion analysis

5×10^4^ EGFP-SKBR3, 2.5×10^4^ AT-MSCs alone, and 5×10^4^ SKBR3 cells mixed with 2.5×10^4^ AT-MSCs (ratio 2:1) were plated in the wells of 24-well plates and cultured in 2 ml of complete culture medium for two days. Cell-free supernatants were collected and subjected to human Bio-Plex™ 27-plex Cytokine Assay (Bio-Rad Laboratories Inc, Hercules, CA). Measurements were performed on Luminex 100 System (Luminex Corporation, Austin, TX) in duplicates with two different AT-MSCs isolates. Results were expressed as mean pg/ml of culture medium ± SD.

In order to confirm the SDF-1α secretion SDF1-α Quantikine Immunoassay (R&D Systems Inc.) was used. SDF-1α levels in cell free supernatants were determined on xMark™ Microplate Spectrophotometer (BIO-RAD).

### Cell proliferation

The effect on tumor cell proliferation was evaluated as a relative fluorescence determined by green fluorescence readout (Ex. 485, Em. 520) on PolarStar OPTIMA reader (BMG Labtechnologies, Offenberg, Germany) in direct cocultures. Quadruplicates of 1×10^4^ EGFP-SKBR3 cells were seeded in black-walled 96-well plates (Greiner Bio-One Intl. AG) with increasing numbers of AT-MSCs and cultured for 6 days. Green fluorescence was directly proportional to the number of viable tumor cells within the wells and the fluorescence value in the untreated cells was set to 100% by default. Experiments were evaluated as mean of quadruplicates ± SD.

In order to dissect the role of SDF-1α/CXCR4 axis in proliferation of EGFP-SKBR3 cells in cocultures with AT-MSCs, specific inhibitor of this signaling axis AMD 3100 (Sigma, St. Louis, MO) was used. Final concentration of 5 μg/ml AMD 3100 was added to EGFP-SKBR3 cells alone, cultured in MSC-CM or in coculture with AT-MSCs. The effect on proliferation was evaluated as a relative fluorescence as described above.

Relative cell viability was evaluated by CellTiter-Glo™ Luminescent Cell Viability Assay (Promega Corporation, Madison, WI) based on the ATP quantitation representative of metabolically active cells. Quadruplicates of 6×10^3^ SKBR3 cells per well were seeded in 96-well plates overnight. Diluted MSCs-CM was added to the adherent tumor cells on the next day. Relative proliferation was determined on LUMIstar GALAXY reader (BMG Labtechnologies, Offenburg, Germany). Values were expressed as mean relative luminescence ± SD, when luminescence of control cells was taken as reference. Experiments were repeated at least twice with similar results and a representative result is shown.

### Chemosensitivity

Following drugs were used: 5-fluorouracil (5FU, Sigma, St. Lois, MO), doxorubicin (DOX, EBEWE Pharma, Austria) and cis-platin (EBEWE Pharma, Austria). For the evaluation of chemosensitivity, either 6×10^3^ EGFP-SKBR3 cells alone or mixed with AT-MSCs (ratio 2:1) were seeded in 96-well plates. On day 0, treatments were started with doxorubicin (6.25 -100 ng/ml), 5FU (6.25-1000 ng/ml) or cis-platin (0.001-10 μg/ml). The chemosensitivity was determined by fluorescence measurements as described above 6 days later. Experiments were evaluated as means of three different experiments run in quadruplicates and the relative fluorescence in untreated cells was taken as 100% by default. Alternatively, 8×10^3^ EGFP-SKBR3 were seeded in 96-well plates overnight and treated with the drugs diluted in MSCs-CM. Relative fluorescence and cell proliferation was determined as above.

### Caspase-3/7 assay

Quadruplicates of 2×10^4^ SKBR3 per well were seeded in 96-well white-walled plates (Corning Costar Life Sciences, Amsterdam, NL) overnight. Doxorubicin (100 ng/ml) or 5FU (100 μg/ml and 500 μg/ml) diluted in MSC-CM or culture media was added to the cells for the indicated period of time and a Caspase-3/7 activity was determined by the Caspase-Glo® 3/7 Assay (Promega Corporation, Madison, WI) on LUMIstar GALAXY reader (BMG Labtechnologies, Offenburg, Germany) at indicated timepoints. Values were determined as mean values of RLU ± SD.

### Annexin V assay

In order to quantify a proportion of viable, apoptotic and necrotic cells in cocultures, adherent AT-MSCs were labeled with 5 μM carboxy-fluorescein diacetate, succinimidyl ester (CFDA-SE, Molecular Probes, Eugene, OR) in a serum-free DMEM for 15 min at 37°C. Medium was replaced for standard culture medium to incubate overnight. Next day, SKBR3 cells were mixed with CFDA-SE labeled AT-MSCs in a ratio 2:1 and plated onto 6-well plate (5×10^4^ SKBR3, 5×10^4^ AT-MSCs, or 5×10^4^ SKBR3 with 2.5×10^4^ AT-MSCs/well) for direct co-culture. Doxorubicin at final concentration 50 ng/ml was added to the respective wells one day later and cells were treated for 48 hrs. Apoptotic cells were stained with Phycoerythrin-labeled Annexin V (eBioscience, San Diego, CA); dead cells were detected with DAPI viability dye. Cells were analyzed using BD CantoII cytometer (Becton Dickinson, USA) equipped with FACSDiva program. FCS Express software was used for the evaluation.

### Statistical analysis

Studies involving comparison between the two groups were analyzed by an unpaired Student's t-test in GraphPad Prism® software (LA Jolla, CA). The value of p < 0.05 was considered statistically significant.

## Results

### AT-MSCs stimulate an EMT and mammosphere formation in the breast cancer cells SKBR3

**Figure 1:**
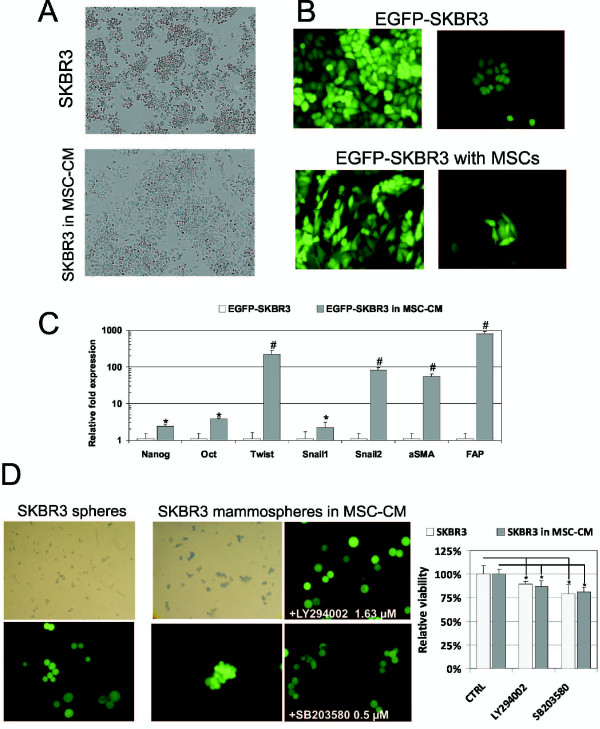
**AT-MSCs induced epithelial–to-mesenchymal transition and increased mammosphere formation. A)** SKBR3 cells were cultured in DMEM or MSC-CM for 6 days. Phase-contrast images revealed shift from epithelial to mesenchymal-like morphology in the majority of tumor cells (magnification 40x). **B)** AT-MSCs and EGFP-SKBR3 were directly cocultured for 6 days. Fluorescence microscopy confirmed morphological signs of an EMT in the tumor cells (magnification 100x). **C)** Markers of the EMT and pluripotency were up-regulated in the tumor cells cultured in MSC-CM. Quantitative RT-PCR confirmed significant increase in the expression of Nanog, Oct, Twist, Snail1, Snail2, αSMA and FAP in EGFP-SKBR3 cells exposed to MSC-CM in comparison to EGFP-SKBR maintained under the standard culture conditions. The data are expressed as means ± SD, *p < 0.05, ^#^p < 0.001. **D)** Non-adherent culture conditions increased EGFP-SKBR3 mammosphere formation in the presence of MSC-CM, which could be abrogated by specific inhibitors of PI3K (1.63 μM LY294002) and p38 MAP kinases (0.5 μM SB203580) (magnification: light microscopy 40x, fluorescent microscopy 100x). Relative viability of EGFP-SKBR3 cells exposed to MSC-CM in the presence of inhibitors did not significantly differ from the viability in DMEM as evaluated by luminescent viability assay (right panel).

Previously we have described that AT-MSCs secrete a plethora of chemokines and growth factors which might affect the tumor cell behavior [23]. When SKBR3 cells were maintained in MSC-CM morphological changes in the majority of tumor cells could be observed (Figure 1A). Very similar effect could be observed in the EGFP-SKBR cells directly cocultured with the AT-MSCs for 6 days (Figure 1B). Cells shifted from the epithelial-like cobble-stone morphology to the spindle-like fibroblastoid appearance. EGFP-SKBR3 cells acquired mesenchymal-like phenotype that resembled an epithelial-to-mesenchymal transition with scattered colony appearance and increased adherence. Up-regulation of the EMT-associated markers in MSC-CM exposed EGFP-SKBR3 cells was confirmed (Figure 1C). MSC-CM treated tumor cells exhibited significantly higher expression of EMT regulators TWIST, Snail1, Snail2, related genes αSMA (α-smooth muscle actin) and fibroblast-activating protein (FAP) in comparison to unaffected EGFP-SKBR3 cells. The EMT process was previously linked to contribute to increased stemness [35] and an upregulation of Oct and Nanog was also detected in MSC-CM exposed EGFP-SKBR3 (Figure 1C). Paracrine factors secreted by AT-MSCs also substantially supported SKBR3 mammosphere formation (Figure 1D). We hypothesized that it was due to stimulation of signaling pathways downstream of receptor-tyrosine kinases by MSCs secretome. Indeed, the pharmacological inhibition of phosphatidylinositol-3-kinase (PI3K) with specific inhibitor LY294002 (1.63 μM) or p38 mitogen-activated protein (MAP) kinase with inhibitor SB203580 (0.5 μM) prevented mammosphere formation in MSC-CM. The viability of SKBR3 in MSC-CM and standard culture conditions was decreased to the same extent by these inhibitors (Figure 1D, right panel).

### Paracrine signaling and migration of SKBR3 cells is influenced by AT-MSCs

**Figure 2:**
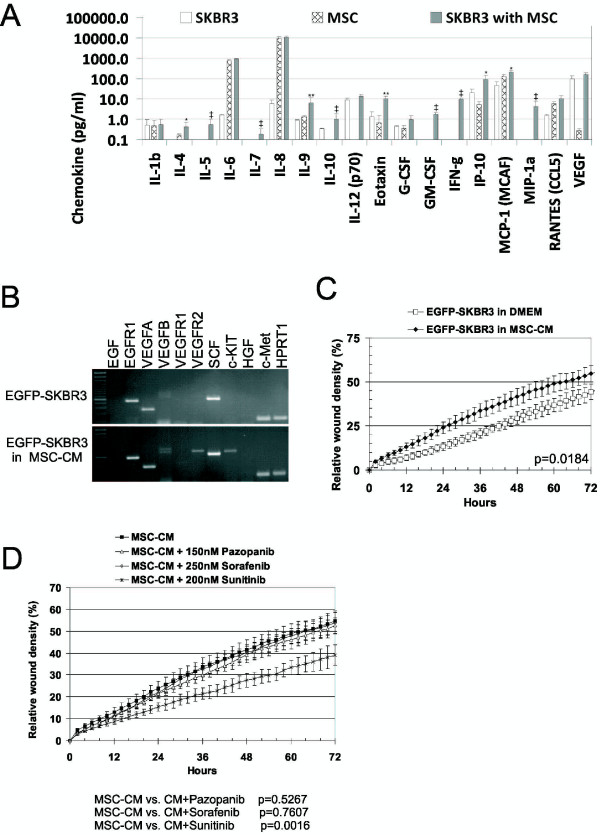
**Increased migration of breast cancer cells due to the changes in a paracrine signaling and gene expression in MSC-CM-exposed SKBR3. A)** Paracrine signaling in tumor and stromal cell cocultures. AT-MSCs were directly cocultured with EGFP-SKBR3 for 2 days and the cytokine levels were evaluated by human Bio-Plex^TM^ 27-plex Cytokine Assay. Direct coculture of the tumor and stromal cells resulted in induction of IL-5, IL-7, IL-10, GM-CSF, IFN-γ and MIP-1a (‡). EGFP-SKBR3 or AT-MSCs alone did not produce detectable levels of these cytokines. Levels of cytokines IL-4, IL-9, eotaxin, IP-10, MCP-1 were significantly higher in comparison to the theoretically calculated additive value of EGFP-SKBR3 and AT-MSCs alone. Values were calculated as means of two independent experiments performed in duplicates, *p < 0.05, **p < 0.01. **B)** Expression analysis demonstrated the induction of VEGFR2 and c-Kit receptor expression in MSC-CM exposed EGFP-SKBR3 cells for 6 days. The expression of EGFR1, VEGFA, SCF and c-Met was detected in EGFP-SKBR3 cells. Induced expression of VEGFR2 and c-Kit was detected in MSC-CM cultured EGFP-SKBR3. Representative outcome is shown; the experiments were repeated at least three times with different MSCs isolates and similar outcome. **C)** EGFP-SKBR3 cells exhibited increased migration in the presence of MSC-CM as evaluated by live-cell imaging in a scratch wound assay. Confluent monolayers of EGFP-SKBR3 cells were wounded and the migration in the presence of MSC-CM or standard culture medium was observed for 72 hrs. Quantitative evaluation of a relative wound density demonstrated the capability of the secreted soluble factors by AT-MSCs to significantly increase the migration of tumor cells. Data are expressed as means of three independent measurements each run in quadruplicates ± SD. **D)** Increased migration of EGFP-SKBR3 in MSCs-CM could be significantly inhibited by 200 nM Sunitinib (VEGFR2, PDGFRβ and c-Kit inhibitor); and not by 150 nM Pazopanib (multi-target kinase inhibitor of VEGFR1, VEGFR2, VEGFR3, PDGFR, FGFR, c-Kit and c-Fms) or 250 nM Sorafenib (VEGFR-2, Raf-1 and B-Raf inhibitor).

In order to further characterize the intercellular crosstalk, we analyzed a cytokine secretion pattern in the SKBR3-MSCs cocultures (Figure 2A). Detectable levels of IL-5, IL-7, IL-10, GM-CSF, IFN-γ and MIP-1a could be measured in the medium from the cocultured cells. These chemokines were below detectable level in the SKBR3 or MSC-CM medium. Moreover, IL-4, IL-9, eotaxin, IP-10 and MCP-1 levels were synergistically increased in the cocultures. Furthermore, the expression of several other growth factors and their cognate receptors was examined as these were previously implicated to play a role in the mutual tumor-stroma interplay [12,28,36]. MSC-CM induced the expression of both c-Kit (stem cell factor receptor) and VEGFR2 receptors in MSC-CM exposed SKBR3 cells (Figure 2B). These data suggested that the interaction of the tumor and stromal cells resulted in altered composition of secreted molecules and expression pattern of the tumor cell.

As it was previously suggested the MSC also affected the tumor cell migration [19]. We could confirm significantly increased migration of MSC-CM-exposed SKBR3 in a wound healing assay as well (Figure 2C). The role of upregulated VEGFR2 or c-Kit signaling in the increased migration of MSC-CM-exposed SKBR3 was further examined by its pharmacological inhibition with multi-target kinase inhibitors Sunitinib (VEGFR2, PDGFRβ and c-Kit inhibitor), Sorafenib (VEGFR-2, Raf-1 and B-Raf inhibitor) and Pazopanib (multi-target kinase inhibitor of VEGFR1, VEGFR2, VEGFR3, PDGFR, FGFR, c-Kit and c-Fms). The migration of SKBR3 in MSC-CM was significantly decreased with 200 nM Sunitinib; and did not change in 150 nM Pazopanib or 250 nM Sorafenib (Figure 2D). These data reflect the differential properties of these inhibitors and a capability of sunitinib to revert MSC-CM-stimulated migration of SKBR3 cells. In accordance with these data, HGF/c-Met signaling was excluded to contribute to increased migration because the expression level of HGF and c-Met did not change and a specific inhibitor of this signaling axis SU11274 did not suppress MSC-CM stimulated SKBR3 migration (data not shown).

### AT-MSCs inhibit proliferation of breast cancer cells SKBR3

**Figure 3:**
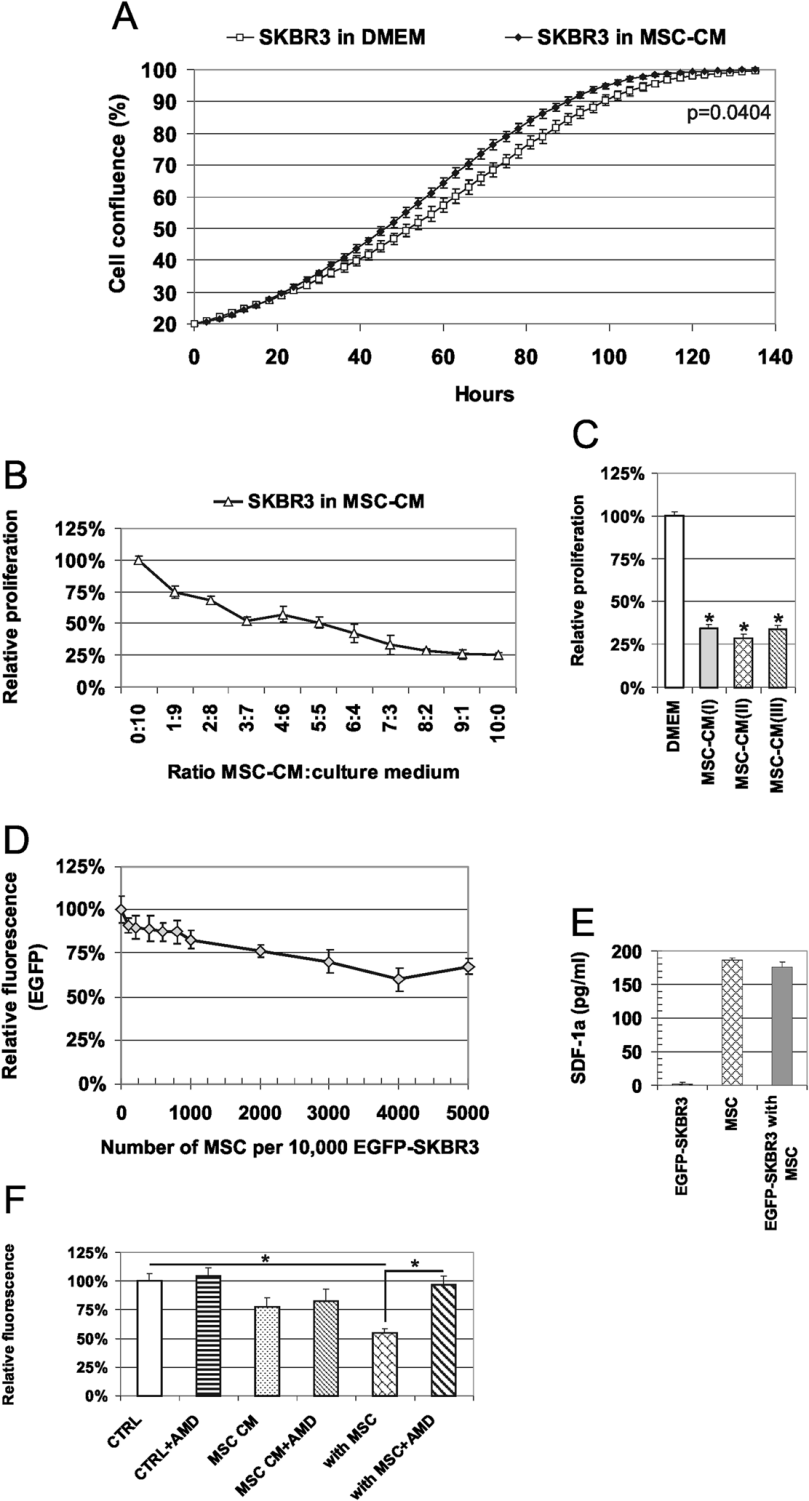
**AT-MSCs and MSC-CM can inhibit the proliferation of SKBR3 breast cancer cells. A)** MSC-CM-exposed EGFP-SKBR3 cells show significantly increased relative confluence as determined by the kinetic live-cell imaging. Data were pooled from the three independent experiments and expressed as means ± SD. **B)** Relative proliferation of the SKBR3 cells in serially diluted MSC-CM was determined by the viability luminescence-based assay after 6 days. MSC-CM supplemented culture medium gradually decreased the cell proliferation in comparison to the standard culture conditions. Proliferation was significantly inhibited in each MSC-CM dilution in comparison to standard culture medium (p < 0.05). **C)** The inhibition of proliferation was determined for the three different AT-MSCs isolates tested as above, *p < 0.05. **D)** Direct coculture of the EGFP-SKBR3 with AT-MSCs confirmed the inhibition of tumor cell proliferation based on the decrease in relative green fluorescence corresponding to the signal from the viable tumor cells. Relative proliferation was significantly lower in comparison to the proliferation of EGFP-SKBR3 alone when ≥ 1,000 AT-MSCs were admixed to the 10,000 EGFP-SKBR3 cells (p < 0.05). **E)** Immunoassay confirmed the production of SDF-1α in the AT-MSCs and cocultures of AT-MSCs and EGFP-SKBR3. **F)** EGFP-SKBR3 cells were cultured in the presence of AT-MSCs CM or AT-MSCs with or without 5 μg/ml AMD3100 - specific inhibitor of SDF1α/CXCR4 signaling. Relative tumor cell viability was significantly lower in the presence of AT-MSCs and the inhibitory effect was abrogated in the presence of the AMD3100. Each experiment was performed at least three times in quadruplicates with similar results and one representative outcome is shown. Data were expressed as means ± SD. *p < 0.05.

Tumor cell proliferation is frequently affected by stromal cells; and therefore we evaluated the effect of AT-MSCs on SKBR3 proliferation. Kinetic-life cell imaging unraveled significantly increased relative confluence of MSC-CM exposed EGFP-SKBR3 (Figure 3A). This was due to the altered morphology and increased cell adhesion of the tumor cells with mesenchymal-like appearance due to EMT (Figure 1A-B). The proliferation of tumor cells was substantially inhibited both in the MSC-CM supplemented cultures (Figure 3B-C) and the direct cocultures with AT-MSCs (Figure 3D). MSCs-mediated anti-proliferative effect was dose dependent and observed with each AT-MSCs isolate examined. Based on the previous reports by the group of P. Rameshwar [21,37], we hypothesized that CXCR4/SDF-1α could be involved in AT-MSCs mediated proliferation inhibition. We confirmed that the AT-MSCs and SKBR3/AT-MSC cocultures secreted SDF-1α (Figure 3E). Therefore we examined whether the pharmacological inhibition of signaling by AMD3100 (a selective inhibitor of CXCR4/SDF-1α signaling axis) would be able to abrogate anti-proliferative effect of AT-MSCs. EGFP-SKBR3 proliferation in 5 μg/ml AMD3100 in the presence of AT-MSCs returned back to the value of cells in direct cocultures without inhibitor (Figure 3F) in spite of the low CXCR4 expression in SKBR3 cells [38,39]. No significant effect of the AMD3100 was observed in the MSC-CM exposed SKBR3 cells, indicating the role of other paracrine factors in MSC-CM mediated inhibition of tumor cell proliferation.

### SKBR3 chemosensitivity is altered in the presence of MSC-CM or AT-MSCs

**Figure 4:**
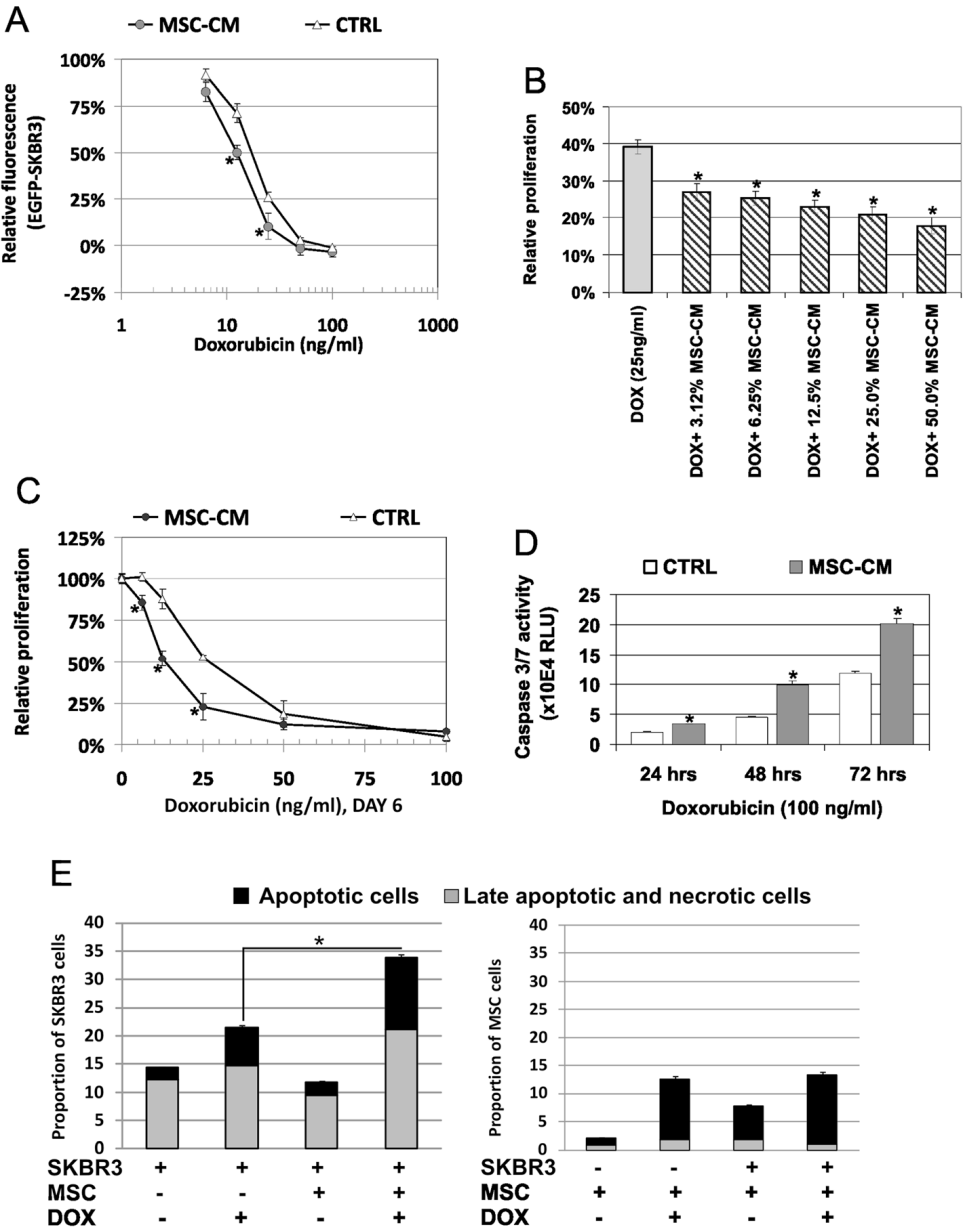
**Increased chemosensitivity of SKBR3 in MSC-CM or AT-MSCs cocultures. A)** Doxorubicin decreased relative fluorescence of the EGFP-SKBR3 in the presence of MSC secreted factors, indicative of increased chemosensitivity of EGFP-SKBR3 cells in the MSC-CM to doxorubicin concentrations of 12.5 ng/ml and 25 ng/ml, *p < 0.05. **B)** Relative viability of MSC-CM-exposed tumor cells is significantly lower in the presence of 25 ng/ml doxorubicin in comparison to doxorubicin treatment in culture medium, *p < 0.05. **C)** Direct comparison of the doxorubicin sensitivity revealed shift in IC_50_(SKBR3) = 27 ng/ml DOX to IC_50_(SKBR3 in MSCs-CM) = 13 ng/ml DOX, as determined by luminescent viability assay, *p < 0.05. **D)** Cytotoxic treatment with doxorubicin induced significantly higher Caspase-3/7 activation in the SKBR3/MSC-CM cultures as determined by a Casp-3/7 luminescence assay. Each experiment was performed three times with four different MSCs isolates and one representative evaluation is shown. Data are expressed as means ± SD, *p < 0.05. **E)** Flow cytometric analysis of the directly cocultured cells unraveled significantly increased proportion of apoptotic and necrotic tumor cells as determined by the Annexin V and/or DAPI positivity in cocultures. Tumor cells were mixed with AT-MSCs (2:1) and treated with 50 ng/ml doxorubicin for 48 hrs. Representative data derived from one experiment were shown, *p < 0.05.

**Figure 5:**
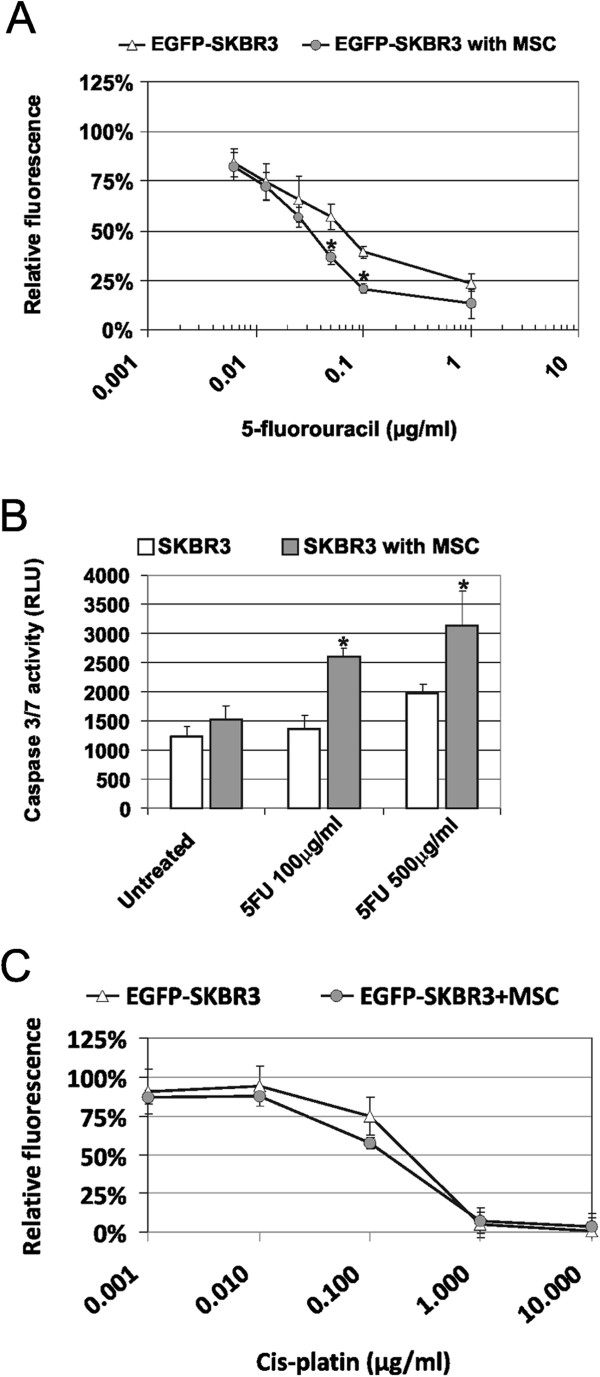
**AT-MSCs affect chemoresistance to 5FU in the direct cocultures with SKBR3 cells. A)** AT-MSCs were directly cocultured with EGFP-SKBR3 in the presence or absence of 6.25 ng/ml - 1 μg/ml 5FU for 6 days. Relative viability of EGFP-SKBR3 was determined by fluorescence measurements. The presence of the AT-MSCs did not interfere with the fluorescence signal. AT-MSCs significantly decreased the resistance to 12.5 ng/ml and 50 ng/ml 5FU. IC_50_ shifted from IC_50_(SKBR3) = 70 ng/ml 5FU to IC_50_(SKBR3 in MSCs-CM) = 35 ng/ml 5FU, *p < 0.05. **B)** AT-MSCs significantly increased Caspase3/7 activation in SKBR3 cells in response to 5FU treatment for 48 hrs. No Caspase 3/7 activity was induced in AT-MSCs cell under these conditions due to their inherent chemoresistant nature, *p < 0.05. **C)** AT-MSCs did not significantly affect sensitivity of tumor cells to cis-platin treatment. Data were derived from the three independent experiments performed in quadruplicates. Values were expressed as means ± SD, p > 0.05.

Next, we decided to analyze whether the AT-MSCs influenced the chemosensitivity of EGFP-SKBR3 cells to anti-cancer drugs such as doxorubicin (DOX), 5-fluorouracil (5FU) and cis-platin. Initial evaluation revealed significantly decreased relative fluorescence of EGFP-SKBR3 cells in response to 12.5 ng/ml and 25 ng/ml doxorubicin diluted in MSC-CM (Figure 4A). Increase in the cytotoxicity of 25 ng/ml doxorubicin correlated to the increasing MSC-CM concentration (Figure 4B). Soluble factors present in MSC-CM decrased the IC_50_ value for doxorubicin in SKBR3 cells twofold: IC_50_(SKBR3) = 27 ng/ml DOX was shifted to IC_50_(SKBR3 in MSC-CM) = 13 ng/ml DOX as determined by the luminescent viability assay (Figure 4C) due to significantly increased apoptosis in the doxorubicin treated tumor cells in the presence of MSC-CM (Figure 4D). Same effect could be also confirmed in the direct SKBR3-AT-MSC cocultures treated with 50 ng/ml doxorubicin for 48 hrs by flow cytometric measurements (Figure 4E). Viability of doxorubicin-treated AT-MSCs did not significantly change in coculture (86.0% vs. 84.5%) as expected. The viability of SKBR3 cells after doxorubicin treatment shifted from 79.9% to 67.5% in the presence of AT-MSCs. Furthermore, the treatment of EGFP-SKBR3 cells with 6.25 ng/ml, 12.5 ng/ml or 25 ng/ml 5FU in the presence of AT-MSCs significantly increased cytotoxicity as measured by the viability assay (Figure 5A). IC_50_ shifted from IC_50_(SKBR3) = 70 ng/ml 5FU to IC_50_(SKBR3 in MSCs-CM) = 35 ng/ml 5FU in the direct cocultures. 100 μg/ml and 500 μg/ml 5FU induced significantly higher Caspase-3/7 activation in SKBR3 cells in the presence of MSCs (Figure 5B). These 5FU concentrations did not induce any cytotoxicity or significantly increased Caspase3/7 activity in AT-MSCs as published previously [33]. Chemosensitivity of EGFP-SKBR3 cells to 0.001-10 μg/ml cis-platin was not significantly changed in the presence of AT-MSCs (Figure 5C).

## Discussion

MSCs represent multipotent cells valuable for regenerative therapies including augmentation of tissue regeneration in breast reconstruction after cancer-related surgery. Although recent results suggested that AT-MSCs might improve a long-term retention of the grafts, the risks of this cellular treatment still remain unresolved specifically in the context of a patient with cancer history [5,6]. Tumors always encompass both malignant part and non-malignant cells of various cell lineages with complex mutual interactions between particular cell types [2,40]. MSCs can contribute to the tumor microenvironment and play a role in mammary carcinogenesis [11]. Our data showed that AT-MSCs did not increase the proliferation of the HER2-overexpressing, estrogen/progesterone receptor negative breast cancer cells SKBR3. However, AT-MSCs induced an EMT in tumor cells with increased tumor cell migration and mammosphere formation, potentially leading to increased aggressiveness and metastatic capability. MSCs derived from bone marrow were already described to affect breast cancer cell proliferation, migration, invasiveness, metastasis, morphology, chemoresistance and hormone responsiveness (reviewed in [11,41]). According to our data the MSCs can alter tumor biology regardless of their tissue origin. Similarities in the MSCs secretome dictate the nature of the interaction with the other cell types [9]. It has been shown that a gene expression profile of the MSCs derived from breast adipose tissue is comparable to the MSCs originating from abdominal adipose tissue resulting in comparable stimulation of proliferation in breast cancer cells MCF7 and MDA-MB-231 [42]. Moreover, the MSCs from primary breast cancer tissues were also shown to exert stimulatory effect on MCF7 proliferation and tumor growth [43]. Detailed study of migration properties of the tumor-cell exposed MSCs have unraveled increased migration of the MSCs isolated from breast adipose tissues in comparison to the migration of the MSCs derived from abdominal adipose tissue [44]. Gene expression profile of these migratory MSCs was close to the profile of MSCs isolated from the tumor-adjacent breast adipose tissues [44]. Thus the MSCs derived from abdominal adipose tissue with lower responsiveness to tumor-induced motility might be preferred exogenous cell source for fat grafting and breast augmentation to limit the effect on mammary carcinogenesis.

MSCs-secreted cytokines induced an EMT, increased expression of pluripotency genes and mammosphere formation in breast cancer cells (Figure 1C-D and [24]) which might suggest the capability of MSCs to increase the proportion of tumor initiating cells as a consequence of the EMT [35,45]. MSC-CM induced expression of VEGFR2 concomitant with high VEGFA expression in SKBR3 cells could generate autocrine loop directly affecting a tumor cell survival and potentially more invasive phenotype [46]. Based on these data, we hypothesized that SKBR3 cells in combination with AT-MSCs might have increased tumorigenicity. However, no increase in the tumor-forming capabilities was observed when AT-MSCs were coinjected with EGFP-SKBR3 cells *in vivo*. AT-MSCs could not support the xenotransplant growth in immunodeficient mice (data not shown). The EMT and upregulation of pluripotency genes induced by MSC-CM was not sufficient to promote tumor growth in low tumorigenic SKBR3 cells*.* Recently Karnoub's group demonstrated that the MSCs-mediated EMT was neither sufficient nor necessary for a generation of cancer stem cell phenotype, although it contributed to the increased metastasis *in vivo*[47]. Future studies will be focused on the attempt to develop tumor xenotransplant model to test the MSCs-mediated alterations in the tumor behavior and its chemosensitivity *in vivo*.

Our data further support the dual role of MSCs in tumor cell proliferation. Previously we have reported increased proliferation of breast cancer cells T47D, MCF7 and MDA-MB-361 in response to AT-MSCs [19,23] in contrast to antiproliferative action on SKBR3 cells (Figure 3). Our data correspond with the findings by Donnenberg *et al.,* who did not show the capability of the AT-MSCs to increase the proliferation of dormant tumor cells [6]. Several studies reported that the MSCs could actually inhibit tumor growth *in vivo*[29,48-50] although in different tumor types (glioblastoma, leukemic, thyroid and colon cancer cells). More importantly, substantially altered composition of the chemokine secretome in tumor-stromal coculture indicated how an inflammatory component of the tumor might arise *in vivo*[51,52]. IP-10 (chemokine CXCL10) is an important mediator in bidirectional MSCs/breast cancer signaling [53]. Its increase in the normoxic conditions and different AT-MSCs/SKBR3 coculture model further extends its importance in stromal/breast cancer interactions.

MSCs were also suggested to contribute to altered tumor drug resistance [21,22,54,55]. Recently the study by Roodhart *et al.* demonstrated that cis-platin-preexposed MSCs mediated systemic resistance to cis-platin in tumor models including breast cancer cells MDA-MB-231 [22]. However our experiments indicated that soluble factors present in the MSC-CM or the AT-MSCs concomitantly exposed to chemotherapeutic drug in direct coculture were not able to mediate chemoresistance (Figures 4 and 5). SKBR3 tumor cells in the presence of AT-MSCs had significantly increased sensitivity to chemotherapeutic drugs doxorubicin and 5FU that are frequently used for the breast cancer treatment. No significant difference in sensitivity to cis-platin (Figure 5C) or paclitaxel (data not shown) was detected when the AT-MSCs and tumor cells were exposed to the drug in cocultures. We believe that a concomitant exposure of stromal and tumor cells to the drug might actually increase the treatment efficiency. Contrastingly the exposure of (circulating) MSCs to the chemotherapy might induce secretion of mediators which subsequently contributed to increased tumor cell resistance [22,55]. It remains to be further evaluated, which mechanisms are drug-specific, tumor cell type-specific or context specific. Taken together the mutual tumor/stromal interactions do not only determine the biological behavior of tumor as a complex organ, but also its response to the chemotherapeutic treatment. The effects of MSCs on tumor cells are multiple and depend on the state of the tumor cell (dormant vs. actively-proliferating), the properties of specific MSCs populations, and interactions with other cell types, such as tumor infiltrating immune cells origin [56]. It is important to focus on the evaluation of interactions of MSCs with primary tumor cells to shed more light into the operating interactions and signaling pathways.

## Conclusions

The aim of our study was to analyze biological effects of AT-MSCs on breast cancer cells SKBR3. We have demonstrated that AT-MSCs induced morphological changes, epithelial-to-mesenchymal transition, increased adherence, mammosphere formation, migration and decreased proliferation in SKBR3. These features and mechanisms of bidirectional signaling are shared by the MSCs originating from adipose tissue with the bone-marrow derived MSCs and considered to play an important role in the breast cancer pathogenesis. Our results indicated the capability of AT-MSCs and secreted soluble factors to increase the chemosensitivity of SKBR3 cells to doxorubicin and 5-fluorouracil. We concluded that the MSC-mediated influence on the drug resistance is dependent on the context of treatment, its timing and a cell type. Based on our observations, we concluded that the tumor and stromal cells interacted in a complex fashion that altered the properties of tumor cells and created dynamic interaction relevant for the tumor behavior and responses.

## Abbreviations

5FU: 5-fluorouracil; αSMA: α-smooth muscle actin; AT-MSCs: Adipose tissue-derived mesenchymal stromal cells; CCL5: Chemokine (C-C motif) ligand 5, RANTES; c-Kit: Stem cell factor receptor; c-MET: Hepatocyte growth factor receptor; CXCR4: Chemokine (C-X-C) motif receptor 4, CXCL12 receptor; DOX: Doxorubicin; EGF: Epidermal growth factor; EGFP: Enhanced green fluorescent protein; EGFR: EGF receptor; EMT: Epithelial-to-mesenchymal transition; FAP: Fibroblast activating protein; FGF: Fibroblast growth factor; GAPDH: Glyceradehyde-3-phosphate dehydrogenase; G-CSF: Granulocyte-colony stimulating factor; GM-CSF: Granulocyte monocyte-colony stimulating factor; HGF: Hepatocyte growth factor; HPRT1: Hypoxanthine phosphoribosyltransferase 1; IFNg: Interferon γ; IL: Interleukin; IP-10 (CXCL10): Chemokine (C-X-C motif) ligand 10; MAP: Mitogen-activated protein; MCP-1 (CCL2): Monocyte chemoattractant protein-1, chemokine CCL2; MIP-1a (CCL3): Macrophage inflammatory protein-1alpha; MSC-CM: Mesenchymal stromal cell-conditioned medium; MSCs: Mesenchymal stromal cells; PDGF: Platelet derived growth factor; PI3K: Phosphoinositol-3’-phosphate kinase; POU5F1 (Oct-3/4): POU factor class 5 homeobeox 1; RANTES (CCL5): Regulated on Activation, Normal T-cell Expressed and Secreted, chemokine CCL5; SCF: Stem cell factor; SDF-1α: Stromal cell-derived factor-1α, CXCL12; TAF: Tumor associated fibroblasts; VEGF: Vascular endothelial growth factor; VEGFR: VEGF receptor.

## Competing interests

The authors declare that they have no competing interests.

## Authors’ contribution

Concept, design and development of methodology: LK, MM, MB; acquisition of data LK, SS, MM, ZK; analysis and interpretation of data LK, MM, SS, ZK; writing of the manuscript and review: LK, SS; technical and material support: MB. All authors read and approved the final manuscript.

## Pre-publication history

The pre-publication history for this paper can be accessed here:

http://www.biomedcentral.com/1471-2407/13/535/prepub

## Supplementary Material

Additional file 1: Figure S1 **A)** AT-MSCs immunophenotype was evaluated by MSC immunophenotyping kit (Miltenyi Biotec, Cologne, Germany). The AT-MSCs were consistently and stably positive for the CD73, CD90 and CD105 (>90%). **B)** Differentiation capabilities of the AT-MSCs were examined by previously established protocols. Adipogenic differentiation was preformed in differentiation media composed of αMEM medium supplemented with 15% FBS, 0.5 μM hydrocortisone, 0.5 mM isobutylmethylxanthine, and 60 μM indomethacin for 28 days. Cells were washed with PBS, fixed in 4% formalin for 1 hr, and stained for 15 min with fresh Oil Red O solution (Fisher Scientific, Hampton, NH). Osteogenic differentiation was performed in osteogenic medium using Osteogenic stem cell kit (StemCell Technologies, Grenoble, France) as recommended by manufacturer. Medium αMEM was supplemented with 15% human osteogenic stimulatory supplements, 10^-8^ M dexamethasone, 0.2 mM ascorbic acid, 10 mM β-glycerolphosphate. Medium was replaced every 3–4 days for 28 days. Cultures were washed with PBS, fixed in 4% formalin for 1 hr, and stained for 10 min with 1 ml of 40 mM Alizarin red (pH 4.3). Osteogenic differentiation was confirmed by detection of red stained calcium deposits. Chondrogenic differentiation was performed by manufacturer’s protocol using human StemMACS ChondroDiff Media (Miltenyi Biotec, Cologne, Germany) and toluidine blue staining. Trilineage differentiation capacity of the AT-MSCs was confirmed. Click here for file

Additional file 2: Table S1 Primer sequences. Click here for file
