# Supplementary material for: Altered features and increased chemosensitivity of human breast cancer cells mediated by adipose tissue-derived mesenchymal stromal cells
Source: BMC Cancer. 2013 Nov 9;13:535. doi: 10.1186/1471-2407-13-535 (PMC3829110; doi:10.1186/1471-2407-13-535)
Supplement: Additional file 2: Table S1 — Primer sequences. [file 1471-2407-13-535-S2.doc]

**Supplementary table**

**Table 1.** Primer sequences

| **Gene** | **Primer sequences (5´→ 3´)** | | | | | | | **Annealing Temp. (°C)** | **Size (bp)** |
| --- | --- | --- | --- | --- | --- | --- | --- | --- | --- |
| **aSMA** | 5- GCACCCCTGAACCCCAAGGC-3 (sense) | | | | |  | | 60 | 136 |
|  | 5- GCACGATGCCAGTTGTGCGT-3 (antisense) | | | | |  | |  |  |
| **CCL5** | 5- CCTCGCTGTCATCCTCATT-3 (sense) |  |  |  |  | |  | 54 | 97 |
|  | 5- GGCAATGTAGGCAAAGCA-3 (antisense) |  |  |  |  | |  |  |  |
| **c-Kit** | 5-GCCCACAATAGATTGGTATTT-3 (sense) | | | | |  | | 60 | 570 |
|  | 5-AGCATCTTTACAGCGACAGTC-3 (antisense) | | | | | | |  |  |
| **c-MET** | 5- CAGATGTGTGGTCCTTTG-3 (sense) |  |  |  |  | |  | 54 | 110 |
|  | 5- ATTCGGGTTGTAGGAGTCT-3 (antisense) |  |  |  |  | |  |  |  |
| **EGF** | 5- AGCAATTGGTGGTGGATG-3 (sense) | | | | |  | | 60 | 103 |
|  | 5- ACTCTTTGCAAAAGTTGTC-3 (antisense) | | | | |  | |  |  |
| **EGFR1** | 5- GAGAGGAGAACTGCCAGAA-3 (sense) |  |  |  |  | |  | 58 | 454 |
|  | 5- GTAGCATTTATGGAGAGTG-3 (antisense) |  |  |  |  | |  |  |  |
| **FAP** | 5- GGTGGATGGTCGAGGAACAGC-3 (sense) | | | | |  | | 60 | 169 |
|  | 5- TCCTCCATAGGACCAGCCCCA-3 (antisense) | | | | | | |  |  |
| **HGF** | 5-CACGGAAGAGGAGATGAGAA-3 (sense) |  |  |  |  | |  | 54 | 120 |
|  | 5 – AAAATCATCCAGGACAGCAG-3 (antisense) |  |  |  |  | |  |  |  |
| **HPRT1** | 5- TGACACTGGCAAAACAATGCA-3 (sense) | | | | |  | | 62 | 136 |
|  | 5-GGTCCTTTTCACCAGCAAGCT-3 (antisense) | | | | |  | |  |  |
| **NANOG** | 5- GCAAATGTCTTCTGCTGAGATGC -3 (sense) |  |  |  |  | |  | 60 | 207 |
|  | 5-AGCTGGGTGGAAGAGAACACAG-3 (antisense) |  |  |  |  | |  |  |  |
| **PDGF-BB** | 5- GAAGGAGCCTGGGTTCCCTG-3 (sense) | | | | |  | | 60 | 232 |
|  | 5-TTTCTCACCTGGACAGGTCG-3 (antisense) | | | | |  | |  |  |
| **POU5F1**  **(OCT)** | 5- GAGTGAGAGGCAACCTGGAGAA-3 (sense) |  |  |  |  | |  | 60 | 226 |
|  | 5- GCCAGAGGAAAGGACACTGGT-3 (antisense) |  |  |  |  | |  |  |  |
| **SCF** | 5-ACTTGGATTCTCACTTGCATTT-3 (sense) | | | | |  | | 60 | 505 |
|  | 5-CTTTCTCAGGACTTAATGTTGAAG-3 (antisense) | | | | | | |  |  |
| **SNAI1** | 5- CAACTGCAAATACTGCAACAAGGA-3 (sense) | | | | | | | 60 | 245 |
|  | 5- ACTTCTTGACATCTGAGTGGGTCTG-3 (antisense) | | | | | | |  |  |
| **SNAI2** | 5- CTTTTTCTTGCCCTCACTGC-3 (sense) |  |  |  |  | |  | 60 | 224 |
|  | 5- GCTTCGGAGTGAAGAAATGC-3 (antisense) |  |  |  |  | |  |  |  |
| **TWIST1** | 5- CATCCTCACACCTCTGCATTCT-3 (sense) | | | | |  | | 60 | 255 |
|  | 5- ACTATGGTTTTGCAGGCCAGTT-3 (antisense) | | | | | | |  |  |
| **VEGFA** | 5- ATCACGAAGTGGTGAAGTTC-3 (sense) |  |  |  |  | |  | 60 | 265 |
|  | 5- TGCTGTAGGAAGCTCATCTC-3 (antisense) |  |  |  |  | |  |  |  |
| **VEGFB** | 5- CCATGAGCCCTCTGCTCCGCC-3 (sense) | | | | |  | | 58 | 678 |
|  | 5- GCCATGTGTCACCTTCGCAGC-3 (antisense) | | | | | | |  |  |
| **VEGFR1** | 5- GAAGGCATGAGGATGAGAGC-3 (sense) |  |  |  |  | |  | 60 | 324 |
|  | 5- CAGGCTCATGAACTTGAAAGC-3 (antisense) |  |  |  |  | |  |  |  |
| **VEGFR2** | 5- GTCAAGGGAAAGACTACGTTGG-3 (sense) | | | | |  | | 58 | 591 |
|  | 5- AGCAGTCCAGCATGGTCTG-3 (antisense) | | | | |  | |  |  |
